# Prevalence and associated factors of self-reported lifetime epilepsy symptoms in an older population in Russia: the Ural Very Old Study

**DOI:** 10.3389/fneur.2026.1751674

**Published:** 2026-02-06

**Authors:** Mukharram M. Bikbov, Gyulli M. Kazakbaeva, Ellina M. Iakupova, Leisan I. Gilemzianova, Anastasiia V. Insapova, Diana A. Timerbulatova, Songhomitra Panda-Jonas, Jost B. Jonas

**Affiliations:** 1 Ufa Eye Research Institute of Bashkir State Medical University, Ufa, Russia; 2 Privatpraxis Prof. Jonas und Dr. Panda-Jonas, Heidelberg, Germany; 3 Department of Ophthalmology, University Hospital Heidelberg, Heidelberg, Germany; 4 Rothschild Foundation Hospital, Institut Français de Myopie, Paris, France; 5 LV Prasad Eye Institute, Hyderabad, Telangana, India; 6 Singapore Eye Research Institute, Singapore National Eye Center, Singapore, Singapore; 7 Beijing Visual Science and Translational Eye Research Institute (BERI), Beijing Tsinghua Changgung Hospital, Tsinghua Medicine, Tsinghua University, Beijing, China; 8 Department of Ophthalmology and Visual Sciences, The Chinese University of Hong Kong, Hong Kong, Hong Kong SAR,China; 9 New York Eye and Ear Infirmary of Mount Sinai, Icahn School of Medicine at Mount Sinai, New York, NY, United States

**Keywords:** neck pain, population-based study, Russia, self-reported lifetime epilepsy symptoms, Ural Very Old Study

## Abstract

**Objective:**

The study aimed to explore the prevalence of self-reported lifetime epilepsy symptoms (SLESs) and associated factors among an elderly population in Russia.

**Methods:**

The population-based Ural Very Old Study (UVOS) was conducted in Bashkortostan, Russia. Of 1882 eligible inhabitants aged 85 + years, 1,526 (81.1%) participated, including 389 (25.5%) men and 1,137 (74.5%) women. The participants underwent a detailed medical examination and interview, during which a history of epileptic attacks was assessed using standardized questions.

**Results:**

Of the 1,526 participants, 1,523 (99.9%), with a mean age of 88.8 ± 2.9 years (range: 85.0–103.1 years), provided information on epilepsy-related questions during the interview. A history of self-reported lifetime epilepsy symptoms was found in 23 individuals (1.5%; 95% confidence interval (CI): 0.90, 2.12), including 4 (17%) men. The mean age of these individuals was 89.2 ± 3.8 years (85.0–103.1 years). In a multivariable analysis, a higher prevalence of self-reported lifetime epilepsy symptoms was associated with a higher prevalence of neck pain (OR:3.53;95%CI:1.35,9,22;*p* = 0.01), iron deficiency-related anemia (OR: 5.68; 95%CI: 1.66, 19.5; *p* = 0.006), and unconsciousness (OR: 7.5295%CI: 2.69, 21.0; *p* < 0.001), as well as with a lower prevalence of any alcohol consumption (OR:0.14;95%CI:0.05, 0.44;*p* < 0.001) and a higher erythrocyte sedimentation rate (OR: 1.04; 95%CI: 1.01, 1.08; *p* = 0.01). It was not associated with level of education (*p* = 0.67), sex (*p* = 0.54), region of habitation (*p* = 0.18), Russian versus non-Russian ethnic background (*p* = 0.48), prevalence of diabetes (*p* = 0.93), or stages of arterial hypertension (*p* = 0.85).

**Conclusion:**

Self-reported lifetime epilepsy symptoms, experienced at any period of life and assessed using a questionnaire with standardized questions, were reported by 23 of 1,523 participants (1.5%) in this very old population in Bashkortostan. The prevalence was independent of sex, educational level, ethnic background, rural versus urban region of habitation, diabetes mellitus, and arterial hypertension.

## Introduction

Epilepsy is one of the most common central nervous system disorders worldwide, affecting individuals of all ages (1–3). Characterized by recurrent, unprovoked seizures due to abnormal, excessive, or synchronous neuronal brain activity, it is associated with up to a threefold increased risk of premature death (4). Owing to the substantial economic, psychosocial, physical, and mental burden experienced by affected individuals, epilepsy has been identified by the World Health Organization as a top priority for the prevention and control of non-communicable diseases (5). To reduce the deleterious effect of epilepsy, knowledge about its prevalence is essential for initiating evidence-based actions and awareness campaigns, as well as for strengthening public and private efforts to improve the quality of and access to care. While the Global Burden of Disease Study (GBD) recently summarized global, regional, and national data on the prevalence of epilepsy, information on epilepsy prevalence in Russia and Central Asia remains scarce. For global summary meta-analyses, data for these regions have often been interpolated (6–8). Therefore, the purpose of our study was to estimate the prevalence of epilepsy, defined as self-reported lifetime epilepsy symptoms (SLESs), in a very old, multi-ethnic population in a region of Russia.

## Methods

As described recently, the Ural Very Old Study (UVOS) is a population-based investigation. Its urban component was conducted in the Kirovskii district of Ufa, the capital of the Republic of Bashkortostan, Russia. Its rural component was conducted in the Karmaskalinsky district, approximately 65 km from Ufa (9–24). The study protocol was approved by the Ethics Committee of the Academic Council of the Ufa Eye Research Institute (protocol number 3; dated 10 August 2017), and all participants provided written informed consent. It was confirmed that all methods were carried out in accordance with the relevant guidelines and regulations. With a multi-ethnic population of approximately 4 million, Bashkortostan is located in the Volga district in the southwest of the Ural Mountains. Recruitment and examinations for the study began on 28 November 2017 and concluded on 5 December 2020. The inclusion criteria were age 85 years or older and residence in the study areas.

The study population consisted of 1526 individuals, with women (*n* = 1136) comprising the majority (74.4%) of the cohort. The study population comprised 81.1% of the total group of 1882 eligible individuals. Participation rates did not differ significantly between the rural and urban components (81.3% vs. 80.2%). According to the 2021 Russian census, the UVOS cohort did not differ markedly from the general population of Russia in terms of sex and age distribution (25).

As described in detail in previous reports, social workers visited the study participants in their homes and conducted a standardized, detailed interview comprising more than 300 questions on a wide range of topics, such as socioeconomic background, self-reported ethnicity, educational level, diet, smoking habits, alcohol consumption, physical activity, quality of life, quality of vision, history and symptoms of major internal medical conditions, previous injuries, interpersonal violence, cognitive function, and hearing loss. The questions were drawn from standardized instruments, such as the Center for Epidemiologic Studies Depression Scale (CES-D), the Folstein test, the Zung Self-Rating Depression Scale, the National Eye Institute Visual Function Questionnaire-25 (VFQ-25), the Questionnaire for Verifying Stroke-Free Status (QVSFS) from the American Heart Association, and the Michigan Neuropathy Screening Instrument (26–39). In particular, the interview included questions regarding symptoms of epilepsy: (1) Have you ever had attacks of shaking in the arms or legs that you could not control? (2) Have you ever had attacks in which you fell and became pale? (3) Have you ever lost consciousness? (4) Have you ever had attacks in which you fell and lost consciousness? (5) Have you ever had attacks in which you fell and bit your tongue? (6) Have you ever had attacks in which you fell and lost control of your bladder? (7) Have you ever had brief attacks of shaking or trembling in one arm or leg or in the face? (8) Have you ever had attacks in which you lost contact with the surroundings and experienced abnormal smells? (9) Have you ever been told that you have, or have had, epilepsy or epileptic fits? (Tables 1, 2) (40). If the study participants were cognitively unable to respond to the questions, the interviewers obtained information from other family members regarding previous epileptic episodes. Epilepsy was defined as present if both questions #1 and #2 were affirmative, or if any of the questions #5 to #9 were affirmative (40). Due to their non-specificity for epilepsy, questions #3 and #4 were primarily not taken into account for the definition of epilepsy. However, answers to these questions were compared with responses to the other questions in the statistical analysis.

**Table 1:** Questions related to the prevalence of self-reported lifetime epilepsy symptoms in the Ural Very Old Study.

Specific questions asked in the interview	*n*	Percentage of the total study population	95% confidence interval
Question #1: Have you ever had attacks of shaking in the arms or legs that you could not control? (no/yes)	79	5.2%	4.2, 6.2
Question #2: Have you ever had attacks in which you fell and became pale? (no/yes)	14	0.9%	0.0, 1.9
Question #3: Have you ever lost consciousness? (no/yes)	134	8.8%	7.3, 10.3
Question #4: Have you ever had attacks in which you fell and lost consciousness? (no/yes)	19	1.2%	0.2, 2.2
Question #5: Have you ever had attacks in which you fell and bit your tongue? (no/yes)	2	0.1%	0.0, 0.1
Question #6: Have you ever had attacks in which you fell and lost bladder control? (no/yes)	4	0.3%	0.0, 0.8
Question #7: Have you ever had brief attacks of shaking or trembling in one arm or leg or in the face? (no/yes)	13	0.9%	0.4, 1.3
Question #8: Have you ever had attacks in which you lost contact with the surroundings and experienced abnormal smells? (no/yes)	6	0.4%	0.0, 0.9
Question #9: Have you ever been told that you have, or have had, epilepsy or epileptic fits? (no/yes)	6	0.4%	0.0, 0.9
Questions #1 and #2 answered “Yes,” or any of Questions #5 through #9 answered “Yes.”(no/yes)	23	1.51%	0.90, 2.12
Questions #1 and #2 answered “Yes.”(no/yes)	9	0.59%	0.21, 0.98
Any of questions #5 through #9 answered “Yes.”(no/yes)	18	1.18%	0.6, 1.73

**Table 2:** Univariate regression analysis of sociodemographic and clinical factors associated with the prevalence of self-reported lifetime epilepsy symptoms in Russia.

Factor	Odds ratio	95% confidence interval of the odds ratio	*P*-value
Age (years)	1.05	0.92, 1.20	0.46
Sex (male/female)	1.64	0.56, 4.85	0.37
Region of habitation (rural/urban)	1.10	0.37, 3.26	0.86
Ethnicity (non-Russian/Russian)	1.33	0.58, 3.04	0.51
Body height (cm)	1.05	0.99, 1.11	0.13
Body weight (kg)	1.01	0.97, 1.06	0.63
Waist circumference (cm)	0.96	0.85, 1.09	0.54
Hip circumference (cm)	0.98	0.94, 1.02	0.30
Waist/hip circumference ratio	40.8	0.28, 6,043	0.15
Waist/height ratio	0.07	0.00, 50.5	0.42
Level of education (1–8)	0.99	0.81, 1.21	0.91
Socioeconomic score	0.95	0.79, 1.14	0.57
Current smoking status (no/yes)	0.15	0.02, 1.20	0.07
Alcohol consumption, any (no/yes)	0.35	0.14, 0.90	0.03
Number of daily meals	0.94	0.56, 1.56	0.80
In a week, how many days do you eat fruits?	0.89	0.73, 1.09	0.27
In a week, how many days do you eat vegetables?	0.93	0.72, 1.21	0.59
Type of cooking oil used: vegetable cooking oil/animal fat (butter)	0.81	0.21, 2.41	0.71
Consumption of whole-grain foods (no/yes)	0.00	0.00	0.99
Salt consumed per day (g)	0.80	0.64, 1.01	0.06
Degree of meat processing (weak/medium/strong)	1.48	0.69, 3.15	0.31
Number of cups of coffee per day	1.27	0.78, 2.07	0.35
Number of cups of tea per day	0.81	0.54, 1.21	0.31
History of angina pectoris (no/yes)	5.00	1.81, 13.8	0.002
History of asthma (no/yes)	25,293,372	–	0.998
History of arterial hypertension (no/yes)	1.15	0.43, 3.13	0.78
History of arthritis (no/yes)	3.23	1.36, 7.67	0.008
History of bone fractures (no/yes)	1.33	0.58, 3.04	0.51
History of low back pain (no/yes)	2.56	0.95, 6.94	0.06
History of thoracic spine pain (no/yes)	1.03	0.42, 2.51	0.95
History of neck pain (no/yes)	3.20	1.39, 7.36	0.006
History of headache (no/yes)	5.53	1.87, 16.3	0.002
History of cancer (no/yes)	1.12	0.26, 4.82	0.88
History of cardiovascular disorders, including stroke (no/yes)	1.62	0.63, 4.13	0.32
History of heart attack (no/yes)	0.85	0.25, 2.87	0.79
History of dementia (no/yes)	1.89	0.55, 6.41	0.32
History of diabetes mellitus (no/yes)	0.85	0.20, 3.66	0.83
History of diarrhea (no/yes)	10.9	2.31, 51.3	0.003
History of iron deficiency-related anemia (no/yes)	3.37	1.45, 13.2	0.009
History of low blood pressure requiring hospital admission (no/yes)	6.67	1.47, 30.3	0.01
History of osteoarthritis (no/yes)	1.59	0.62, 4.08	0.33
History of skin disease (no/yes)	3.42	1.14, 10.3	0.03
History of thyroid disease (no/yes)	0.70	0.09, 5.22	0.72
History of unconsciousness (no/yes)	4.87	2.03, 11.7	<0.001
History of menopause (no/yes)			
Age at last regular menstrual bleeding (years)	1.030	0.91, 1.16	0.67
Age at last menstrual bleeding (years)	0.98	0.87, 1.11	0.75
History of falls or injuries (no/yes)	2.74	1.08, 7.00	0.04
Alanine aminotransferase (IU/L)	1.00	0.95, 1.05	0.90
Aspartate aminotransferase (IU/L)	1.01	0.96, 1.05	0.79
Aspartate aminotransferase/alanine aminotransferase ratio	0.92	0.63, 1.34	0.66
Bilirubin, total (μmol/L)	0.98	0.93, 1.05	0.61
High-density lipoprotein (mmol/L)	0.64	0.34, 1.23	0.18
Low-density lipoprotein (mmol/L)	1.03	0.67, 1.59	0.88
Cholesterol (mmol/L)	0.90	0.63, 1.29	0.56
Triglycerides (mmol/L)	1.01	0.54, 1.87	0.98
Rheumatoid factor (IU/mL)	1.15	0.96, 1.37	0.12
Erythrocyte sedimentation rate (mm/hour)	1.03	1.003, 1.06	0.03
Glucose (mmol/L)	0.73	0.47, 1.14	0.17
Creatinine (μmol/L)	0.99	0.97, 1.01	0.39
Estimated glomerular filtration rate (mL/min per 1.73 m^2^)	0.91	0.47, 1.79	0.80
Chronic kidney disease stage	0.52	0.18, 1.56	0.25
Urea (mmol/L)	1.07	0.89, 1.27	0.49
Residual nitrogen (g/L)	18.2	0.09, 3,818	0.29
Total protein (g/L)	1.01	0.95, 1.07	0.71
International normalized ratio (INR)	1.35	0.04, 43.8	0.87
Blood clotting time (minutes)	0.70	0.20, 2.46	0.58
Prothrombin index (%)	1.00	0.95, 1.05	0.98
Hemoglobin (g/L)	0.99	0.96, 1.01	0.23
Erythrocyte count (10^6^ cells/μL)	0.60	0.26, 1.36	0.22
Leukocyte cell count (10^9^ cells/L)	0.91	0.66, 1.27	0.59
Rod-core granulocyte (% of leukocytes)	0.72	0.50, 1.04	0.08
Segment nuclear granulocyte (% of leukocytes)	1.07	1.00, 1.14	0.05
Eosinophil granulocytes (% of leukocytes)	0.58	0.32, 1.03	0.06
Lymphocytes (% of leukocytes)	0.93	0.86, 1.01	0.07
Monocytes (% of leukocytes)	1.04	0.87, 1.24	0.66
Prevalence of diabetes mellitus	0.57	0.13, 2.47	0.45
Anemia (serum hemoglobin concentration <140 g/L in men and <130 g/L in women)	1.79	0.67, 4.73	0.24
Blood pressure, systolic (mm Hg)	1.00	0.99, 1.02	0.84
Blood pressure, diastolic (mm Hg)	0.99	0.96, 1.03	0.72
Blood pressure, mean (mm Hg)	1.00	0.97, 1.03	0.92
Arterial hypertension (defined as stage 1 or higher) (no/yes)	0.89	0.26, 3.07	0.85
Arterial hypertension, stages	1.04	0.67, 1.61	0.85
Ankle-brachial index, right	4.10	0.02, 7,029	0.71
Ankle-brachial index, left	1.70	0.001, 2013	0.88
Prevalence of chronic obstructive pulmonary disease (no/yes)	0.27	0.10, 0.73	0.01
Metabolic syndrome, prevalence (no/yes)	0.84	0.32, 2.17	0.71
StateTrait Anxiety Inventory	1.03	0.99, 1.07	0.14
Depression score	1.03	0.99, 1.07	0.21
Hearing loss score	1.02	0.99, 1.05	0.20
Visual acuity (best-corrected; better eye; logarithm of the minimum angle of resolution (LogMAR))	0.70	0.26, 1.87	0.48
Mild vision impairment in the better eye	0.50	0.07, 3.77	0.50
Moderate to severe vision impairment in the better eye	1.17	0.47, 2.90	0.74
Blindness in the better eye	1.14	0.46, 2.85	0.78

In addition, the participants underwent physical examinations, such as assessment of anthropomorphic parameters and measurement of arterial blood pressure and handgrip strength. We biochemically analyzed blood samples collected under fasting conditions (9–24). The participants also underwent ophthalmological examinations.

The interview was conducted in the participants’ homes. We carried out other examinations at the hospital for individuals who could come to the hospital. Otherwise, we examined them in their homes using portable devices (9–24).

In the statistical analysis, conducted with the help of a software program (SPSS version 29.0; IBM-SPSS Inc., Chicago, United States), we determined the mean value and 95% confidence interval (CI) of the primary outcome parameter—the prevalence of self-reported lifetime epilepsy symptoms. Using binary logistic regression analyses, we examined relationships between SLES prevalence and other parameters, first in a univariate model and subsequently in multivariable analyses. In the multivariable analyses, all variables that showed a significant relationship with the prevalence of SLESs in the univariate analyses were initially included as independent variables. We then removed, in a step-by-step manner, all independent variables that were no longer significantly associated with epilepsy prevalence. We calculated odds ratios (ORs) and their 95% CIs. All *p*-values were two-sided, and statistical significance was defined as a *p*-value of <0.05.

## Results

As reported previously, 1,526 individuals (389 men, 25.5%) participated in the study, representing 81.1% of the 1882 eligible inhabitants. These participants were visited in their homes and completed the interview (9–24, 41). Of these 1,526 individuals, 1,523 (99.9%) provided information on the epilepsy-related questions. Of the 1,526 participants, who were primarily visited in their homes, 105 (6.9%) died after the interview before they could attend the hospital. In addition, 246 (16.1%) individuals did not attend the hospital-based examinations and did not undergo any clinical examinations using portable devices at home. A total of 423 (27.7%) participants did not attend the hospital-based examinations but were clinically examined using portable devices at home, and 751 (49.2%) individuals were examined at the hospital (9–23).

The study cohort comprised 558 (36.3%) individuals of Russian ethnicity, 668 (43.9%) Tatars, 171 (11.2%) Bashkirs, 49 (3.2%) Chuvash, eight (0.5%) Mari, and 69 (4.5%) individuals of other ethnicities. The mean age was 88.8 ± 2.9 years (median: 88.2 years; range: 85–103.1 years).

A history of SLESs was identified in 23 of the 1,523 participants (1.5%; 95% CI: 0.90, 2.12), including 4 men (17%) and 19 women (83%), with a mean age of 89.2 ± 3.8 years (85.0–103.1 years).

In the univariate analysis, higher SLES prevalence was associated with lower prevalence of any alcohol consumption; higher prevalence of a history of angina pectoris, arthritis, neck pain, back pain, diarrhea, and iron deficiency-related anemia, episodes of low blood pressure requiring hospital admission, skin disease, unconsciousness, and previous falls or injuries; lower prevalence of chronic obstructive pulmonary disease; and a higher erythrocyte sedimentation rate (Table 2).

In the multivariable analysis, the following parameters were excluded due to a lack of statistical significance: History of angina pectoris (*p* = 0.68), episodes of low blood pressure requiring hospital admission (*p* = 0.55), history of tumbling (*p* = 0.65), history of arthritis (*p* = 0.56), history of chronic obstructive pulmonary disease (*p* = 0.28), history of back pain (*p* = 0.27), history of skin disease (*p* = 0.06), and diarrhea (*p* = 0.08). In the final model, higher SLES prevalence was associated with higher prevalence of neck pain, iron deficiency-related anemia, and unconsciousness; lower prevalence of any alcohol consumption; and a higher erythrocyte sedimentation rate (Table 3). Even after removing previous unconsciousness from the list of independent variables, higher SLES prevalence remained significantly associated with higher prevalence of neck pain, iron deficiency-related anemia, and unconsciousness, as well as a higher erythrocyte sedimentation rate (Table 3). In that model, SLES prevalence was not associated with socioeconomic or demographic parameters, such as level of education (*p* = 0.67), sex (*p* = 0.54), region of habitation (*p* = 0.18), Russian versus non-Russian ethnic background (*p* = 0.48), prevalence of diabetes (*p* = 0.93), or stages of arterial hypertension (*p* = 0.85).

**Table 3:** Multivariable regression analysis of clinical factors associated with the prevalence of self-reported lifetime epilepsy symptoms in Russia.

Factor	Odds ratio	95% confidence interval of the odds ratio	*P*-value
History of neck pain	3.53	1.35, 9,22	0.01
History of iron deficiency-related anemia	5.68	1.66, 19.5	0.006
History of unconsciousness	7.52	2.69, 21.0	<0.001
Alcohol consumption (beer, whisky, rum, gin, brandy, or other local products)	0.14	0.05, 0.44	<0.001
Erythrocyte sedimentation rate (mm)	1.04	1.01, 1.08	0.01
Excluding history of unconsciousness			
History of neck pain	4.03	1.58, 10.3	0.004
History of iron deficiency-related anemia	6.30	1.92, 20.7	0.002
Alcohol consumption (beer, whisky, rum, gin, brandy, or other local products)	0.21	0.08, 0.60	0.004
Erythrocyte sedimentation rate (mm)	1.04	1.01, 1.07	0.01

## Discussion

In this population-based study of a very old, multi-ethnic population in Southern Russia, the prevalence of SLESs was 1.5% (23 of 1,523 participants). Higher SLES prevalence was associated with higher prevalence of neck pain, iron deficiency-related anemia, and unconsciousness; lower prevalence of any alcohol consumption; and a higher erythrocyte sedimentation rate. It was not associated with level of education, sex, region of habitation, prevalence of diabetes, or stages of arterial hypertension.

SLES prevalence in our very old study cohort corresponds to figures reported previously in other populations (3, 42–45). A recent global and regional meta-analysis conducted by the Global Burden of Disease Study (GBD) estimated that 51.7 million individuals worldwide were affected by epilepsy of any type in 2021, with an age-standardized prevalence of 0.66% (95% CI: 0.57–0.75) (3). Idiopathic epilepsy had a global age-standardized prevalence of 0.31% (9%% CI: 0.24–0.39), while secondary epilepsy had a global age-standardized prevalence of 0.35 (95% CI: 0.32–0.38). In 2021, 0.7% of the global population had active epilepsy, with 0.3% due to idiopathic epilepsy and 0.4% due to secondary epilepsy (3). Epilepsy prevalence was estimated to be markedly higher in low- and middle-income countries. For Russia, the GBD estimated an age-standardized prevalence of 0.50% (95% CI: 0.40–0.59), one of the lowest estimates globally, in contrast to the highest figures reported in some countries of Latin America and the Caribbean (Trinidad and Tobago: 1.37%; 95% CI: 0.95–1.77) and in some sub-Saharan African countries (Gabon: 1.36%; 95%CI: 0.82–1.83). The 0.50% prevalence estimated by the GBD for Russia was markedly lower than the 1.5% found in our cohort. Several factors may explain this discrepancy: The GBD reported age-standardized epilepsy prevalence, while we reported any epilepsy-like event that the study participant could recall; differences in study design (meta-analysis vs. population-based recruitment); differences in the definition and assessment of epilepsy; and differences in the age of the study cohorts. Additional factors may include differences in the ethnic composition of the study cohorts—our study included Russians, Bashkirs, and Tatars—and regional environmental or lifestyle factors specific to Bashkortostan.

Reports on epilepsy prevalence in Russia have been sparse to date. Guekht et al. examined epilepsy prevalence among older adolescents and adults in 14 regions of the Russian Federation, covering a total population of 517,624 individuals in both the European and Siberian parts of the country (6). Based on available medical information sources, the age-adjusted epilepsy prevalence was 0.34% (95%CI: 0.33–0.36), with higher prevalence in men (0.45%; 95%CI: 0.43–0.48) than in women (0.25%; 95% CI: 0.24–0.27) and higher prevalence in the eastern regions of Russia. The highest prevalence was observed in the 50–59-year age group. Other studies from Russia and Central Asia assessed factors associated with epilepsy without reporting detailed prevalence estimates (8). In the study performed by Mil’chakova et al., the age- and sex-standardized epilepsy prevalence was 0.21% (95% CI: 0.18–0.23) in the Soviet region of Makhachkala and 0.30% (95% CI: 0.26–0.34) in the Khasavyurt region of the Republic of Dagestan, with higher prevalence in male individuals (7). A study using regional registry data from the Republic of Bashkortostan for the period 2013–2017 reported an epilepsy prevalence of approximately 0.29–0.33% for adults (46). In the Republic of Bashkortostan, where our study was conducted, a study based on annual reports from neurology services reported an epilepsy prevalence of 0.32% for the period 2013–2017. The mean age of these patients ranged from 30.5 ± 1.3 years to 41.8 ± 0.2 years, and the men/women ratio was 1.1/1.0. The authors noted that the epilepsy prevalence observed in Bashkortostan was higher than the corresponding figure of 0.26% for the Russian Federation as a whole.

In the multivariable analysis, higher SLES prevalence was associated with higher prevalence of neck pain. Although the present cross-sectional study does not allow causal inferences, one may discuss possible mechanisms that could account for the association between SLESs and any common comorbidities, such as shared neurological pathways or musculoskeletal strain during seizures. Shokr et al. highlighted the high-mobility group box 1 (HMGB1) protein as a potentially important factor in this context (47). Similarly, iron deficiency-related anemia was a factor significantly associated with higher SLES prevalence. It can be discussed how systemic metabolic or hematological deficiencies might lower the seizure threshold in a “very old” population, given that iron is an essential cofactor for neurotransmitter synthesis and brain function. In this context, previous studies have revealed that sigma 1 receptors (σ1 receptors) are involved in the neuroprotection and pathophysiology of both conditions and that targeting these receptors may have the potential to modulate both seizures and comorbidities associated with epilepsy, including cognitive impairment (48). Interestingly, study participants with a history of SLESs showed considerably lower prevalence of alcohol consumption overall (OR: 0.14) in the multivariable analysis (Table 2). The cross-sectional design of our study did not allow us to determine whether this association was due to a protective effect or, more likely, a “protopathic bias” resulting from alcohol avoidance among patients with a known diagnosis of epilepsy or those taking anti-seizure medications. In addition, our study could not assess potential interactions between anti-seizure medications and alcohol or lifestyle changes following diagnosis. Finally, a potential inflammatory component was suggested by the correlation between higher SLES prevalence and a higher erythrocyte sedimentation rate (Table 2). It remains unclear whether this association reflects systemic age-related inflammation or chronic low-grade neuroinflammation as a contributing factor to epilepsy and how the pathophysiology of epilepsy in older adults may be related to an elevated erythrocyte sedimentation rate. In this context, Shokr et al. discussed that the sigma 1 receptor, as a multifunctional ligand-activated protein located in the membranes of the endoplasmic reticulum, may mediate a variety of neurological disorders, including epilepsy, amyotrophic lateral sclerosis, Alzheimer’s disease, and Huntington’s disease. It includes neuroprotective effects of sigma 1 receptor agonists by a variety of pro-survival and antiapoptotic sigma 1 receptor-mediated signaling functions (49).

When the findings of our investigation are discussed, the limitations of our study must be considered. First, due to its cross-sectional design, the associations identified in the statistical analysis between SLES prevalence and other variables do not imply causality and may reflect comorbidity, shared risk factors, or consequences of epilepsy or aging. Second, the relatively high age of our study cohort was associated with a risk of survival bias—in the sense that individuals with more severe epilepsy might have died before recruitment—and a risk of a recall bias, that is, difficulty in remembering previous epileptic episodes. However, in the case of cognitively impaired participants, family members were also interviewed about prior epileptic attacks, which likely reduced the impact of this bias on the study results. Third, a major limitation of our study was the definition of epilepsy, which entirely relied on the interview and the standardized questions asked by trained social workers (2, 39, 50). We did not further substantiate the diagnosis through additional clinical examinations, nor did we differentiate between idiopathic and secondary epilepsy. This limitation includes reliance on an exclusively self-reported diagnosis without clinical confirmation, electroretinography, or medical record review, as well as the absence of differentiation between active and remote epilepsy. It also includes the potential misclassification of epilepsy with syncope, falls, or vasovagal events, particularly in very old individuals. Fourth, a substantial proportion of the clinical examinations were conducted in hospital settings or using portable devices, while the epilepsy assessment relied exclusively on interviews. This might have created an asymmetry in validity between the outcome and covariates. Fifth, another limitation of our study is the relatively small number of SLES cases (*n* = 23), which posed a risk of overfitting in multivariable models, including violation of the empirical rule of approximately 10 events per variable. The stepwise elimination of parameters in the creation of the final multivariable model might have inflated associations and increased the instability of the odds ratios. However, more conservative modeling approaches were not feasible due to the low number of cases. Sixth, although the study region was typical of the Volga Federal District, the proportion of Russians in the study cohort was lower than in North-Western and Central Russia. However, since SLES prevalence was independent of ethnic background, it is unlikely that differences in ethnic composition influenced the main findings of our investigation. Seventh, as in any epidemiological study, non-participation may result in selection bias. However, with a participation rate of more than 81%, selection bias was unlikely in our investigation. The strengths of our study include the relatively large sample size of a very old cohort, population-based recruitment of participants, the examination of a wide range of parameters allowing the detection of previously unrecognized associations between SLES prevalence and other variables, and the focus on a region of Russia/Central Asia for which data on epilepsy prevalence have been scarce.

In conclusion, symptoms of epilepsy experienced at any point in life were reported by 23 of 1,523 individuals (1.5%) in a very old population in Bashkortostan, with prevalence independent of sex, educational level, ethnic background, rural versus urban region of habitation, diabetes mellitus, and arterial hypertension.

## Data Availability

The raw data supporting the conclusions of this article will be made available by the authors, without undue reservation.
